# Thulium laser–assisted colorectal endoscopic submucosal dissection

**DOI:** 10.1016/j.vgie.2025.07.013

**Published:** 2025-08-20

**Authors:** Robert Bechara, Mandip Rai

**Affiliations:** Kingston Health Sciences Center, Queen's University, Kingston, Ontario, Canada

## Abstract

**Background and Aims:**

Thulium laser is emerging as an alternative energy source in endourologic applications due to its precise cutting capabilities, reliable hemostasis, and limited thermal injury. These features have promoted its adoption in urology and sparked interest in gastrointestinal endoscopy. In endoscopic submucosal dissection (ESD), thulium laser offers potential advantages over conventional electrosurgical knives, including shallow penetration depth, powerful coagulation, and precise dissection.

**Methods:**

We describe the use of thulium laser–assisted colorectal ESD in a 68-year-old man with a 3.5-cm rectal polyp. Submucosal injection was followed by incision and complete dissection using the thulium laser.

**Results:**

En bloc thulium laser–assisted ESD of the rectal lesion was completed in 75 minutes without the requirement for coagulation forceps. There were no intraoperative or delayed adverse events. The final pathology was tubulovillous adenoma with clear margins.

**Conclusions:**

This case demonstrates the feasibility and potential benefits of thulium laser–assisted colorectal ESD. The precise cutting capabilities, shallow penetration depth, and effective coagulation properties of the thulium laser make it an exciting prospect as an alternative to conventional electrosurgical knives for colorectal ESD. Thulium laser–assisted ESD may offer a safe and effective alternative to conventional knives in colorectal ESD. Larger prospective studies are needed to confirm its safety and efficacy.

## Background

**Figure 1:**
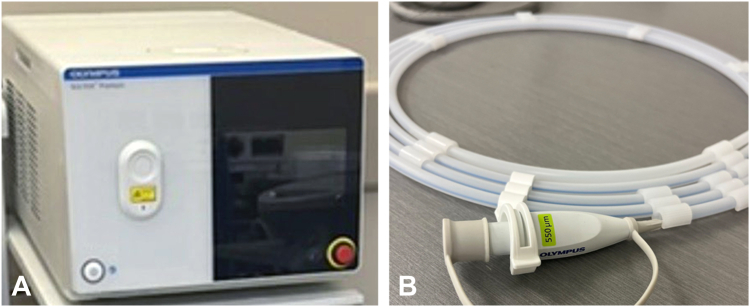
Rectal endoscopic submucosal dissection. **A,** A 3.5-cm Paris Is lesion. **B,** Incision. **C,** Dissection. **D,** Pinned specimen.

Thulium laser is emerging as an alternative energy source in endourologic applications, because of its improved cutting capabilities, reliable hemostasis, and limited thermal injury of adjacent tissue. These features have promoted its adoption in urology for endoscopic enucleation of prostate, bladder tumor resection, and stone management.^1^ The safety, efficacy, and precision observed in endourologic procedures have sparked interest in the use of the thulium laser for gastrointestinal endoscopy (Fig. 1).^2^

In endoscopic submucosal dissection (ESD), thulium laser offers potential advantages over conventional electrosurgical knives. Notably, the laser has a very shallow depth of penetration (∼0.2 mm), which may help reduce the risk of perforation versus traditional electrosurgical knives with a penetration depth of 2 to 3 mm.^3^ It also offers a powerful coagulation effect useful in the dissection of vascular submucosal tissue, as well as a precise target tissue effect that enables exact dissection.

**Figure 2:**
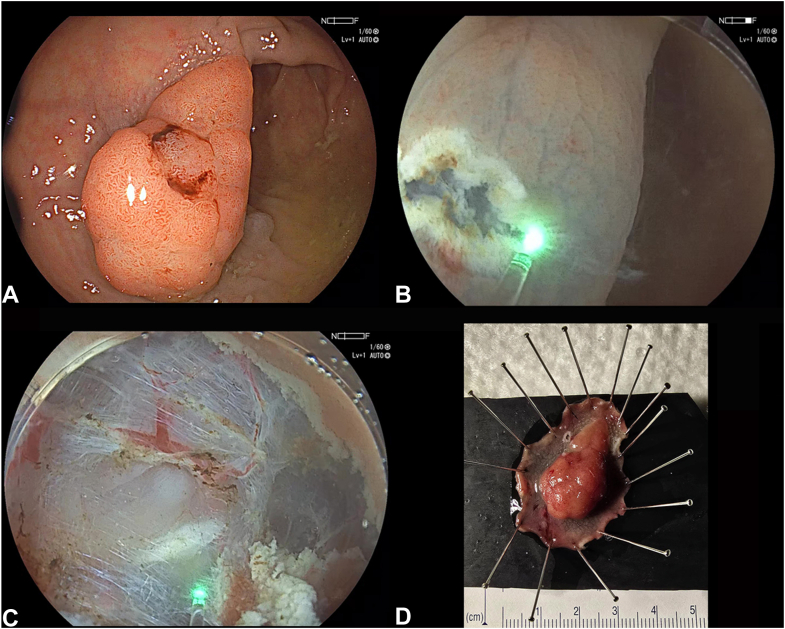
Thulium laser system. **A,** The energy source. **B,** Laser fiber.

Here, we describe the off-label use of the thulium laser system (SOLTIVE SuperPulsed Laser System; Olympus, Tokyo, Japan), which delivers ultrashort pulse widths and precise energy modulation (Video 1, available online at www.videogie.org). This system offers adjustable power settings (2-60 W) and adjustable pulse durations (200 μs to 50 ms), allowing for tailored energy delivery (Fig. 2).

## Case

A 68-year-old man was referred for endoscopic resection of a proximal rectal polyp after a positive fecal immunochemical test result. The polyp was a 3.5-cm Paris Is, Japan Narrow-Band Imaging Expert Team classification 2B, located in the proximal rectum requiring en bloc resection (Fig. 1). ESD was performed with the patient under general anesthesia. To minimize carbonization, an underwater technique was used, analogous to endourologic laser procedures. The laser was set at 0.1 J per pulse, with a frequency of 80 Hz, delivering a total power output of 8 W. Following submucosal injection with hydroxypropyl methylcellulose, a circumferential mucosal incision was made using the thulium laser fiber, and subsequent submucosal dissection was completed. The laser provided precise tissue dissection and reliable hemostasis, without the need for hemostatic forceps. The en bloc ESD was completed in 75 minutes without adverse events. Histopathology was a tubulovillous adenoma with high-grade dysplasia and R0 resection. The patient was discharged the same day and had an uneventful recovery.

## Discussion

This case demonstrates the feasibility of thulium laser–assisted ESD in colorectal lesions. The laser's precise cutting, effective coagulation, and shallow penetration depth contributed to efficient dissection while minimizing thermal injury and perforation risk. Our experience is consistent with that of previous reports, with, to our knowledge, Cho et al^4^ first describing thulium laser use in gastric ESD over a decade ago, and Tontini et al^2^ later demonstrating its efficacy in ex vivo colorectal models. Larger prospective studies are needed to confirm its safety and efficacy for broader application in ESD.

## Patient Consent

Informed consent was obtained from the patient for publication of the case details and accompanying video.

## Disclosure

All authors disclosed no financial relationships.

## Supplementary data

Video 1 Thulium laser technology is emerging as a potential alternative energy source for endoscopic procedures and is already widely used in urology for prostate enucleation and bladder tumor excision. The SOLTIVE SuperPulsed Laser allows for the following: precise cutting, effective hemostasis, and limited thermal damage. Shallow penetration depth (0.2-0.4 mm) theoretically reduces the risk deep tissue injury and perforation. Thulium laser–assisted ESD for gastric neoplasia was first described by Cho et al^4^ >10 years ago. We present a case of a 68-year-old male with a 3.5 cm lesion in the proximal rectum, identified during fecal immunochemical test–positive colonoscopy. The lesion was classified as Paris Is, JNET 2B, necessitating en bloc resection. ESD was performed via Thulium laser–assisted ESD using the SOLTIVE SuperPulsed Laser System. Here we can see a 3.5-cm lesion in the proximal rectum under white light. We then examine it using Linked Color Imaging and Blue Light Imaging, which reveal a central area demonstrating a JNET Type 2B pattern. We proceeded with the mucosal incision using the 550-μm thulium laser. The laser cutting setting was 0.1 J per pulse, with a frequency of 80 Hz, delivering a total power output of 8 W. To minimize carbonization, an underwater technique was employed, similar to approaches used in endoscopic urologic laser surgery. Continuous water irrigation was necessary to maintain a clear view during the incision due to vaporization of the epithelial tissue. One of the differences between thulium laser–assisted ESD and traditional ESD with standard knives is that the thulium fiber is much thinner, allowing for improved suction even through a 2.8-mm channel. Another difference is that, with traditional knives, the tip can be anchored into the tissue, providing stabilization during the cutting movement. Because laser cutting is essentially noncontact, this stabilization is not possible with the current form factor. After completing the circumferential incision, trimming was performed around the incision using the cutting settings. Once this was completed, we applied clip-and-band traction to improve access to the submucosa. With traction applied and improved access to the submucosa, submucosal dissection proceeds more efficiently. Laser energy is applied in short bursts to the submucosa, with constant water irrigation. Note the very shallow depth of laser penetration, making it a safe dissection tool. One drawback of this platform, compared to currently available needle knives, is the lack of ability to perform submucosal injection with the same tool. However, by performing the initial injection with a viscous agent and subsequently using the underwater ESD technique, the need for repeated submucosal injections is minimized Here, we can see penetrating vessels, which traditionally would be precoagulated with coagulation forceps. However, with the excellent hemostatic capabilities of the thulium laser—and using settings of pulse energy 1.5, frequency 40, with a total power of 60 W—this area is easily dissected with excellent hemostasis. Here is the final cut of the remaining submucosa. Note the difference in carbonization between air dissection and underwater technique. The ESD was completed in 75 minutes without complication. The mucosal defect was closed with 13 clips. Final pathology revealed a tubulovillous adenoma with high-grade dysplasia and clear margins. Compared with conventional electrosurgical knives, the thulium laser offers a shallow penetration depth, minimizing deep thermal injury while maintaining excellent coagulative properties. This case demonstrates the feasibility and potential advantages of thulium laser–assisted ESD for colorectal lesions. Further large-scale studies are needed to validate its efficacy in broader patient populations and to define its potential role in gastrointestinal endoscopy.
